# The promotion of pooling level of basic medical insurance and participants’ health: impact effects and mediating mechanisms

**DOI:** 10.1186/s12939-023-01927-1

**Published:** 2023-06-07

**Authors:** Bo Dong

**Affiliations:** School of Political Science and Public Administration, Wuhan University, Wuhan, 430072 Hubei China

**Keywords:** Medical insurance, Provincial pooling, Health, Mediating effect

## Abstract

**Background:**

Enhancing the pooling of basic medical insurance plays a vital role in strengthening the resilience to risk and co-payment capacity of medical insurance funds. In China, there is a concerted effort to shift from municipal to provincial pooling of medical insurance. While existing research suggests that provincial pooling of basic health insurance affects the health of participants, the findings are not yet consistent, and there is limited research on the specific pathways of impact between the two. Therefore, this study aims to explore the influence of provincial pooling of basic medical insurance on participants' health and analyze the mediating role of medical cost burden and medical service utilization.

**Methods:**

Using data from the 2012–2018 China Labor Dynamics Survey (CLDS), this study focuses on a sample of urban workers enrolled in basic medical insurance. After excluding samples with missing information, a total of 5,684 participants were included in the analysis. The effects of the provincial pooling policy of basic medical insurance on participants' medical cost burden, medical service utilization, and health were analyzed using double difference modeling. Furthermore, structural equation modeling was employed to explore the mediating paths between provincial pooling and health.

**Results:**

The findings reveal that provincial pooling of basic medical insurance significantly impacts participants' medical cost burden, medical service utilization, and health. Specifically, provincial pooling helps reduce the participants' medical cost burden (β = -0.1205; *P* < 0.001), improves the level of medical institutions visited (β = 1.7962; *P* < 0.001), and promotes health improvement (β = 1.8370; *P* < 0.001). The mediating effect analysis demonstrates that the direct effect of provincial pooling on health is 1.073 (*P* < 0.001), with a mediating effect of medical cost burden between provincial pooling and health measuring 0.129 (*P* < 0.001). Heterogeneity analysis indicates that provincial pooling is more effective in reducing the burden of medical costs for low-income (β = -0.2273; *P* < 0.001) and high-age participants (β = -0.2710; *P* < 0.001), and it also helps increase the burden of medical costs for low-income (β = 4.0875; *P* < 0.001) and high-age participants (β = 1.9010; *P* < 0.001) based on provider ranking. Moreover, it is found that provincial pooling is more beneficial in improving the health of high-income (β = 1.7984; *P* < 0.001) and middle- and high-age enrollees (β = 1.9220; *P* < 0.001; β = 0.5900; *P* < 0.001). Further analysis reveals that the provincial unified income and expenditure mode has a more positive effect than the provincial risk adjustment fund mode in reducing the medical expense burden of the insured (-0.2053 < -0.0775), improving the grade of medical institutions (1.8552 > 0.8878), and enhancing the health level (2.8406 > 0.6812).

**Conclusion:**

The study concludes that provincial pooling of basic medical insurance has a direct positive impact on participants' health and indirectly promotes health improvement by reducing the burden of medical costs. The effects of provincial pooling on participants' medical cost burden, medical service utilization, and health vary based on income and age. Additionally, the provincial-level unified collection and payment model proves to be more advantageous in optimizing the functioning of health insurance funds through the "law of large numbers" principle.

**Supplementary Information:**

The online version contains supplementary material available at 10.1186/s12939-023-01927-1.

## Introduction

Pooling, corresponding to the concept of fund risk pools, is an important aspect of health insurance systems worldwide. Different types of pools exist based on their size, including no-risk sharing pools, fragmented risk pools, integrated risk pools, and single risk pools [1]. In cases where health insurance fund risk pools are fragmented, consolidation becomes necessary due to challenges such as risk selection and segmentation [2]. Integration, in general, refers to the pooling of funds to diversify risks and provide related services to participants [3]. The level of integration signifies the extent and scope of risk diversification, fund pooling, and service provision, often based on administrative levels within each country.

In the reform of the health insurance system, numerous countries are progressively enhancing the degree of pooling. This is due to the fact that increasing the integration of health insurance funds not only strengthens the funds' resilience to risks and co-funding capabilities but also facilitates the realization of the "law of large numbers" effect in health insurance. For instance, Germany initiated the pooling level reform in 1994 by introducing the risk structure compensation scheme and the central health fund system to the health insurance fund. These measures mitigated the fragmentation of disease funds and bolstered pooling and mutual support among the funds [4].

Similarly, China has undergone a gradual elevation in the level of health insurance pooling. Initially, China implemented a county-level pooling strategy in its health insurance system, resulting in a fragmented state characterized by "regional and sectoral fragmentation". In order to enhance risk diversification within the health insurance fund, China progressed from county-level to municipal-level pooling in its basic health insurance. Subsequently, with the reform of the health insurance system, China commenced the implementation of provincial pooling for basic health insurance funds in 2011, actively promoting the transition from municipal to provincial pooling.

As a significant component of China's healthcare insurance system reform, evaluating the impact of provincial-level reform on the basic medical insurance fund is of utmost importance. Assessing whether it can promote the health improvement of the participants becomes a crucial indicator in measuring the effectiveness of provincial-level medical insurance pooling [5]. Several studies have been conducted to analyze the influence of provincial health insurance pooling on health outcomes, yet consistent conclusions have not been reached. Some investigations have found no substantial changes in patients' health following the transition from municipal to provincial-level health insurance pooling [6], while others have concluded that provincial pooling can significantly enhance participants' health [7].

In summary, provincial health insurance pooling does have an impact on the health of participants, but the divergent findings suggest the need for further analysis of the relationship between the two. Moreover, existing studies have primarily focused on whether provincial pooling affects participants' health without delving into the specific mechanisms involved. Therefore, this study aims to analyze the effects of provincial pooling of basic medical insurance on the health of participants, specifically focusing on the mediating mechanisms, using urban workers' basic medical insurance participants as a case study. This research introduces several innovative aspects compared to existing studies: firstly, it analyzes the pathways through which provincial pooling affects health from two perspectives: medical cost burden and medical service utilization. Secondly, it explores the heterogeneous characteristics of the impact of provincial pooling policies in terms of age and income, providing a more detailed and comprehensive examination of the policy effects. Thirdly, it compares and analyzes the impact of different provincial health insurance pooling models on the health of participants.

## Method

### Policy background

The primary aim of provincial-level pooling of basic medical insurance is to bolster risk resilience and co-funding capacity by managing medical insurance funds on a larger scale. China has been working progressively to strengthen basic medical insurance pooling. By examining policy documents from each province, we summarize the current state of provincial-level basic medical insurance for urban workers in China (Table 1). It reveals that provinces differ in both timelines for achieving increased medical insurance pooling and the specific provincial pooling models.These models can be classified into two types: the provincial collector model and the provincial risk transfer model. The former entails full transfer of medical insurance funds from municipalities to the provincial financial account, forming the provincial pooling fund, while the latter involves transfer of a certain percentage of the funds for reallocating medical insurance balances across different areas. In both cases, municipalities only transfer full (or proportional) amounts of their health insurance fund income to the provincial level, but responsibility for fund collection and supervision remains with municipal health insurance agencies [8].

**Table 1:** Status of implementation of provincial-level co-pooling of basic medical insurance for urban workers in China

Province	Time	Model
Shanghai	2000	provincial-level unified revenue and expenditure model
Beijing	2001	provincial-level unified revenue and expenditure model
Tianjin	2001	provincial-level unified revenue and expenditure model
Tibet	2009	provincial-level unified revenue and expenditure model
Chongqing	2011	provincial-level unified revenue and expenditure model
Hainan	2012	provincial risk adjustment fund model
Ningxia	2017	provincial risk adjustment fund model
Fujian	2019	provincial risk adjustment fund model

In China's medical insurance provincial pooling practices, there are two cases of medical insurance policy changes after implementation. One is the unification of health insurance financing and treatment levels across different regions; the other occurs when differences in health insurance policies among regions persist, and policies remain non-unified. In the unified collection and payment model, health insurance policies across various regions are standardized, and the level of health insurance treatment is enhanced after integration. Under the risk transfer model, there are two types of health insurance policies: one results in the unification of health insurance policies and more balanced benefits across regions, while the other fails to unify policies, thus maintaining disparities in medical insurance treatments. To analyze the mediating role of medical cost burden between provincial pooling and participants' health, our study includes provinces where medical insurance policies are unified, and treatment levels are elevated after provincial pooling—Shanghai, Beijing, Tianjin, Tibet, Chongqing, Hainan, and Ningxia—excluding Fujian, where policies were not unified, and treatment levels were not raised.

### Data source

Our study uses data from the China Labor Dynamics Survey (CLDS), conducted in 2012, 2014, 2016, and 2018 by the Social Science Survey Center of Sun Yat-sen University. CLDS employs a multi-stage, multi-level probability sampling method proportional to labor force size and covers 29 provinces (directly governed municipalities and autonomous regions) in China, thus ensuring national representation. The survey encompasses a broad range of information on basic demographics, socioeconomic status, healthcare utilization, costs, and health for the working-age population (15–64 years old), providing a solid foundation for this study.

Considering China's retirement age (60 years old) and the contribution policy of urban workers' basic medical insurance—which exempts participants from paying medical insurance fees after retirement—we have set the retirement age at 60 and included only participants aged 15–60 in our analysis. To obtain an appropriate sample, several data handling steps were taken: retaining only the basic medical insurance participant samples for urban employees; excluding regional samples with missing information about respondents' city of residence; and removing samples with missing key variable information. The final sample comprises 5,684 valid observations.

### Hypothesis

Theoretically, the following effects of provincial integration of basic medical insurance on participants' health might exist: First, unifying medical insurance policies after raising the level of integration can be considered a more equalizing policy practice [9]. This unification of health insurance financing and reimbursement treatment can lead to a more balanced level of health insurance development among different regions, helping to ensure a steady increase in treatment [10]. The enhanced level of benefits after unification implies lower out-of-pocket costs for participants and a reduction in financial risk, which can improve health by alleviating their medical burden.

Second, raising the level of pooling can remove the original restrictions on medical care access between pooling zones and expand participants' choice of medical care from municipal to provincial levels. This can enhance the accessibility of medical services and increase participants' access to higher-level medical institutions [8]. Although this addresses the demand for medical treatment, it may also encourage unreasonable medical treatment behavior among participants. After basic medical insurance is integrated at the provincial level, management of the medical insurance funds is shifted from the local level to the provincial medical insurance institutions, while fund collection and expenditure supervision responsibilities are still borne by local medical insurance institutions. Once responsibility for balancing health insurance fund income and expenditure is transferred to provincial health insurance institutions, local health insurance institutions have an increased incentive to relax the audit of participants' medical referrals and expense reimbursements, along with supervision of medical institutions' behaviors, to expand fund expenditures and receive more transfer funds from the provincial coordinating fund [7]. Consequently, participants who previously attended lower-level medical institutions may seek higher-level hospitals with more concentrated medical resources and better technology, thereby escalating the moral risk of excessive medical care [11]. Simultaneously, following provincial pooling, reduced motivation of local health insurance agencies to supervise medical institutions will incentivize medical establishments to offer more services to participants, intensifying the moral hazard of demand inducement [12]. In this situation, enrollees' health benefits may not improve due to the moral hazard of both participants and medical institutions.

**Fig. 1:**
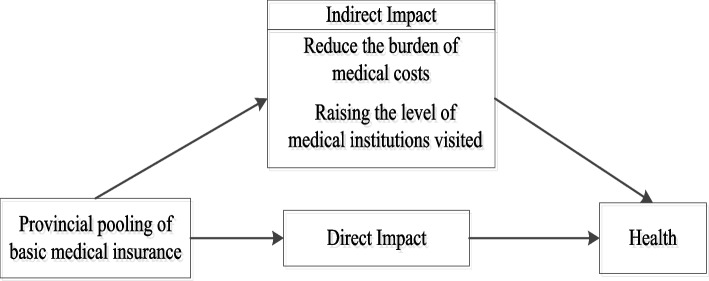
Path analysis of the health impact of provincial pooling of basic medical insurance

Based on this analysis, we propose the following hypotheses to analyze the impact of provincial pooling of basic medical insurance on participants' health and its mediating effect, as illustrated in Fig. 1 of our analytical model:

- Hypothesis 1: Provincial pooling of basic medical insurance can improve participants' health by reducing their medical cost burden.
- Hypothesis 2: Provincial pooling of basic medical insurance can enhance medical institution rankings, but the increase in rankings fails to improve health due to the moral hazard of participants and medical institutions.

### Variables

#### Exogenous variables

This paper examines the impact of upgrading the level of pooling of basic medical insurance from municipal to provincial levels on participants' health and the associated mediating mechanisms. Consequently, the exogenous variables in this study are medical cost burden, medical service utilization, and health. Taking into account that medical cost burden is influenced by household economic conditions and income levels, we employ the ratio of individual out-of-pocket expenses to household income to measure participants' cost burden. Medical service utilization is determined by the level of the medical institution that participants visit. Health is measured using participants' self-rated health, a subjective evaluation and expectation reflecting their overall physical, psychological, and social state [13], commonly used as an indicator in related studies [14]. Participants' self-rated health outcomes span five categories: very unhealthy, relatively unhealthy, fair, healthy, and very healthy, assigned values of 1, 2, 3, 4, and 5, respectively, following Lee's approach [15].

#### Endogenous variables

The key endogenous variable of interest in this paper is the elevation of the level of pooling of basic medical insurance, denoting whether it has shifted from municipal to provincial pooling or if provincial pooling has been implemented. Since the provincial integration of health insurance is promoted on a provincial basis, specific implementation policies are also formulated by each province. To determine whether a province has implemented provincial integration, this paper first obtains policy documents on provincial integration of basic medical insurance through the official websites of provincial governments, health insurance bureaus, or human society bureaus, and obtains the year of policy release based on the dates marked on the documents released by each province regarding the implementation of provincial integration. Subsequently, we match these document release date with the year of the questionnaire survey to code the variable as "1" (indicating provincial integration) or "0" (indicating no provincial integration).

### Control variables

Based on Grossman's health needs model [16], the control variables included in this paper include individual characteristics such as age, gender, education level, marriage, income, and place of regular residence; personal health behavior habits such as smoking and drinking, and whether or not they had a disease in the past two weeks. We also included the level of economic development as a control variable to control for the effect of differences in economic development between provinces on the level of health insurance benefits. According to the data conditions selected, the following were set: age was the difference between the year of interview and the year of birth; gender was a dummy variable, 1 for males and 0 for females; marital status was a dummy variable, 1 if the individual was first married, remarried or cohabiting, and 0 if unmarried, divorced or widowed; education level was a 0–4 level variable based on the individual's education, 0 for never attended school = 0, 1 for primary school = 1, and 1 for primary school = 1. The education level is the 0–4 level variables set according to individual education, which is never attended school = 0, elementary school = 1, junior high school = 2, high school (including technical school and junior college) = 3, university and above = 4: undergraduate (including specialist), postgraduate (master, doctor); income level is the natural logarithm of annual per capita household income; smoking and drinking are dummy variables, yes = 1, representing such habit; no = 0, representing no such habit. The level of economic development is the natural logarithm of GDP per capita. Considering the existence of population mobility, this paper also uses the place of regular residence as a control variable, which refers to the city where the participant lives. We refer to the classification method of the China Statistical Yearbook, which classifies cities into first-class cities, second-class cities, and third-class cities and below. Whether or not the participant was sick in the past two weeks is a measure of the participant's disease status, which is also a dummy variable with yes = 1 and no = 0.Through the above analysis, the variables of this paper are selected and described as shown in Table 2.

**Table 2:** Description of variables

Type	Variables	Definition	
Exogenous variables	Medical cost burden	personal out-of-pocket expenses as a percentage of household income	
	Level of medical institution visited	primary care institutions (clinic/village health office, township health center, community health service center) = 1	
		county and district health institutions = 2	
		provincial city/region/municipality directly under the central government district health institutions = 3	
		provincial/autonomous regions/municipalities directly under the Central Government and above health institutions = 4	
	Health	very unhealthy=1	
		relatively unhealthy=2	
		fair=3	
		healthy=4	
		very healthy=5.	
Endogenous variables	Provincial pooling of basic medical insurance	implementing the policy of provincial pooling of health insurance = 1 no implementation of the health insurance provincial pooling policy = 0	
Control variables	Age	15—29=1	
		30—44=2	
		45—60=3	
	Gender	female = 0	
		male = 1	
	Education	illiteracy = 0	
		elementary school = 1	
		middle School = 2	
		high School = 3	
		university = 4	
	Marriage	no spouse = 0	
		with spouse = 1	
	Income	annual household income per capita	≤30%=1
			31%—60%=2
			≥61%=3
	Smoking Habit	no=0	
		yes=1	
	Drinking habit	no=0	
		yes=1	
	Residence	third-class cities and below=1	
		second-class cities=2	
		first-class cities=3	
	Economic level	per capita GDP	
	Disease	no=0	
		yes=1	

### Study model

Assuming that the treatment and control groups meet the parallel trend assumption, the double-difference method can observe changes in the treatment group before and after the policy, accurately measuring the average before-and-after treatment effect. Through the analysis of the basic, The analysis of the provincial pooling policy of medical insurance in China reveals that eight provinces have already achieved provincial pooling, and there are differences in the time of provincial pooling in different provinces, and the promotion of the provincial pooling policy shows gradual characteristics, which provides the feasibility of this study. Considering the differences in implementation times of provincial integration policy across regions, we adopt the overlapping double-difference method for policy evaluation and employ a panel two-way fixed effects model to test the impact of provincial integration of basic medical insurance on participants' medical service utilization and health. The specific model settings are as follows:

$${Y}_{ict}={\beta }_{1}+{\beta }_{2}{Policy}_{ict}+X'_{ict}\delta +{\mu }_{t}+{\gamma }_{c}+{\varepsilon }_{ict}$$

where the subscripts $i$,$c$, and $t$ denote individual, region, and time, respectively, and the exogenous variable $Y_{ict}$ denotes the health service utilization or health level of individual $i$ in province $c$ in period $t$. The core endogenous variable $Policy_{ict}$ is whether the province $c$ in which individual $i$ is located has implemented provincial pooling of Basic medical insurance for urban workers in period $t$; if provincial pooling is implemented,$Policy_{ict}$ = 1, otherwise 0.In this study, provinces that implemented provincial pooling were the experimental group, specifically including seven provinces, namely Shanghai, Beijing, Tianjin, Tibet, Chongqing, Hainan, and Ningxia. Although Fujian also implemented provincial pooling, it was not included in the analysis of this study because its health insurance policies were not unified. In addition to these provinces, the remaining provinces that did not implement provincial pooling were the control group.$X^{^{\prime}}_{ict}$ is the set of all control variables;$\mu_{t}$ denotes time fixed effects;$\gamma_{c}$ denotes province fixed effects; and $\varepsilon_{ict}$ is a random disturbance term.

In addition, because there are two different models of provincial pooling of basic medical insurance, although both models achieve provincial pooling, there are differences in the scale of their raising and distributing medical insurance funds, which may also affect their effectiveness in protecting the health of participants. Therefore, to compare and analyze the differences between the two models, this paper also further compares and analyzes the differences in the effects of the different models of provincial pooling of health insurance funds.

### Statistical analysis

For data analysis, we used Stata 12.0 for double difference regression analysis, heterogeneity analysis, and robustness test. In the analysis of the mediating role between provincial pooling of basic health insurance and health, AMOS 25.0 was used to build a structural equation model and construct a standardized path test. Bootstrapping was used to test the mediating effects. the test criteria of the structural equation model were the model fitting index, and the specific evaluation criteria were GFI, AGFI, and CFI > 0.9, and RMSEA < 0.08, showing that the model had good validity.

## Results

### The impact of provincial pooling of basic medical insurance on health

Table 3 presents the results of the effects of provincial pooling of basic medical insurance on the medical cost burden, medical service utilization, and health of participants. The results show a negative effect of the policy on the medical cost burden of participants (policy variable coefficient = -0.1205), a positive effect on the level of medical institutions visited by participants (policy variable coefficient = 1.7296), and a positive effect on participants' health (policy variable coefficient = 1.8370). All three effects are significant at the 1% level of significance. These findings suggest that provincial pooling of basic medical insurance reduces the burden of medical costs on participants, enables them to receive services at higher levels of medical institutions, and promotes improvements in their overall health.

**Table 3:** Impact of provincial pooling of basic health insurance on health

Variables	Medical cost burden	Level of medical institution visited	Health
Provincial pooling	-0.1205^c^ (0.0181)	1.7962 ^c^ (0.3573)	1.8370 ^c^ (0.4794)
Age	0.0459 ^c^ (0.0031)	0.0123 (0.0306)	0.1129 ^c^ (0.0354)
Gender	0.0395^b^ (0.0014)	-0.0537 (0.0402)	-0.0348 (0.0363)
Education	0.0005 (0.0007)	0.0493 ^b^ (0.0174)	0.0646 ^c^ (0.0190)
Marriage	0.0003 (0.0018)	-0.0131 (0.0509)	0.0356^a^ (0.0467)
Income	-0.0143 ^b^ (0.0009)	0.0391 (0.0235)	0.0093 ^b^ (0.0229)
Smoking Habit	0.0028^a^ (0.0016)	-0.0104 (0.0414)	-0.0231 (0.0438)
Drinking habit	0.0023 (0.0016)	0.0304 (0.0416)	-0.0124 ^b^ (0.0390)
Residence	0.0481 ^c^ (0.0110)	-0.0541 ^c^ (0.0196)	0.3486 ^b^ (0.0280)
Economic level	-0.0265 (0.0222)	-0.1225 (0.5863)	0.0279 ^b^ (0.3726)
Disease	0.0875 ^c^ (0.0021)	0.0407 ^c^ (0.0339)	-0.3752 ^b^ (0.0554)
Constant	0.2719(0.2563)	3.9821(5.8583)	0.8429(3.9505)
Time fixed effect	Control	Control	Control
Provincial fixed effect	Control	Control	Control
Observation	5684	5684	5684
R^2^	0.8777	0.9115	0.6755

Note: Standard errors are in parentheses; ^c^, ^b^, ^a^ denote significant at the 1%, 5%, and 10% levels of significance

### The mediating path of provincial pooling of basic medical insurance affecting health

In this study, the provincial pooling of health insurance was used as the independent variable, medical cost burden and medical service utilization were used as mediating variables, and participant health was used as the dependent variable. A single-step multiple basic mediation model was set up using AMOS 25.0, its fitness was tested, and the basic model was revised according to the fit results. Table 4 shows the fit indices of the model, where the fitness χ^2^ is 16.536, corresponding to a p-value of 0.168, which is greater than 0.05, indicating that the corrected structural equation model fits the sample data better. In addition, the RMSEA value is 0.029, CFI is greater than 0.90, GFI is greater than 0. 90, and AGFI is greater than 0. 90, indicating that the relevant indices are within a reasonably acceptable range, implying that the overall fit of the data to the model is good and the model can be used for path estimation.

**Table 4:** Model fit index

Evaluation Indicators	Model Results	Adaptation standards	Adaptation judgment
$\chi^{2} /df$	1.378	< 3.00	yes
$\chi^{2}$-valued probability value P	0.168	> 0.05	yes
RMSEA	0.029	< 0.08	yes
CFI	0.985	> 0.90	yes
GFI	0.995	> 0.90	yes
AGFI	0.956	> 0.90	yes
NFI	0.959	> 0.90	yes

**Fig. 2:**
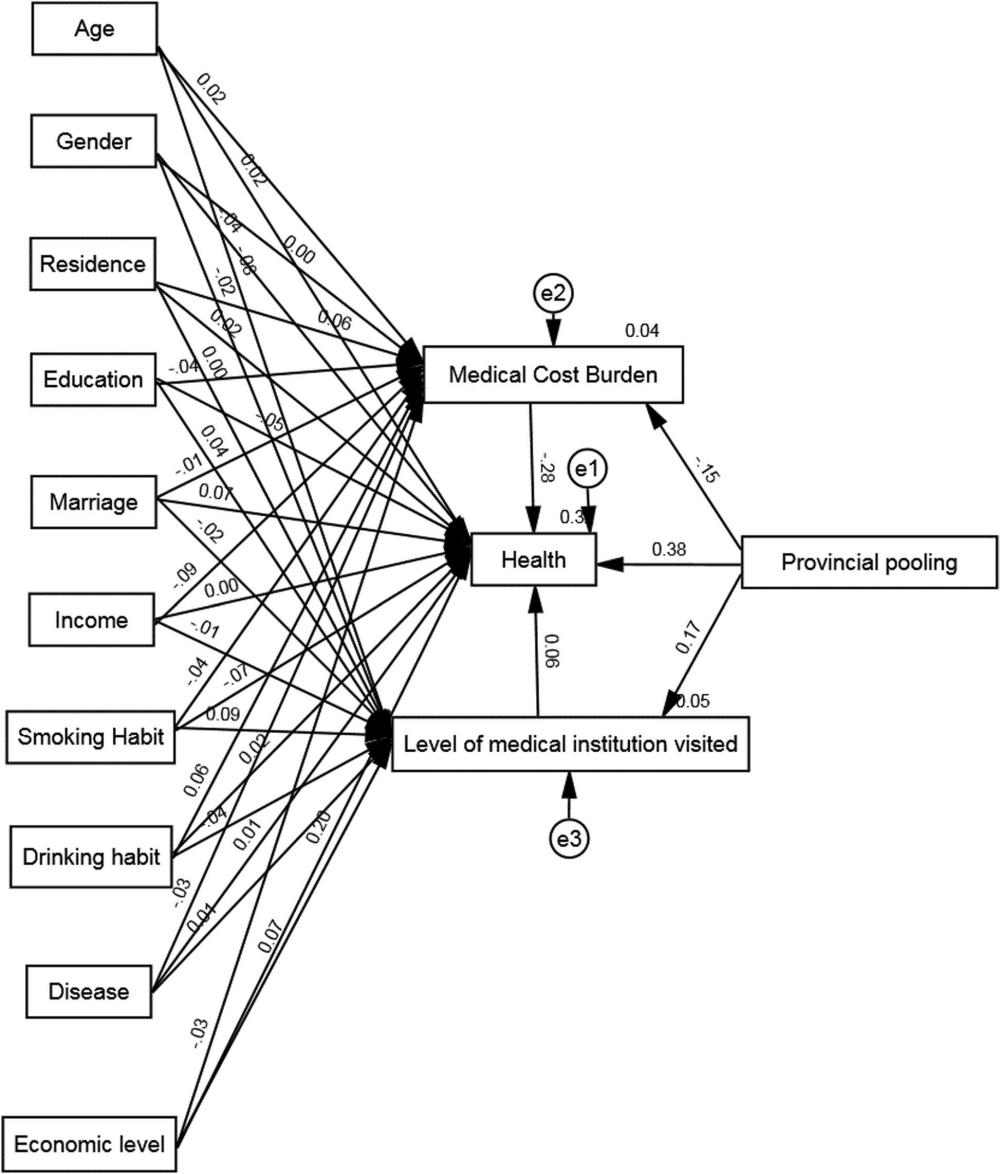
The intermediary pathway of basic health insurance provincial pooling impacting health

To estimate the mediated paths' coefficients, the Bootstrap method was employed, setting 5000 replicate random samples and 95% confidence intervals, using the standard regression coefficients as a judgment criterion. Figure 2 illustrates the path of the effect of provincial pooling of basic medical insurance on health, while Table 5 presents the regression results with medical cost burden and medical institution levels as mediators. The results indicate a significant positive direct effect of provincial pooling of basic medical insurance on participant health with a coefficient of 0.383, and a significant negative effect on medical cost burden with a coefficient of -0.152 at the 1% test level. In contrast, medical cost burden has a significant negative effect on health with a coefficient = -0.276 at the 1% test level, suggesting that the provincial pooling impact on health is mediated through medical cost burden. On the other hand, there is a significant positive effect of provincial pooling on the level of medical institutions visited by participants at the 1% test level, with a coefficient of 0.175. Although the effect of the level of medical institutions visited on health is positive, its standardized regression coefficient does not pass the significance test. Therefore, there is not enough evidence to support the mediating effect between provincial pooling and health related to the increase in the level of medical institutions visited by participants, thus verifying hypothesis 2.

**Table 5:** Results of a mediated pathway test for the health effects of provincial pooling of basic health insurance

PPath	Non-standardized coefficient	Standardization coefficient	SE	CR	P
Provincial pooling → Health	0.944	0.383	0.097	9.756	< 0.001
Provincial pooling → Medical Cost Burden	-0.021	-0.152	0.006	-3.348	< 0.001
Medical Cost Burden → Health	-4.899	-0.276	0.695	-7.051	< 0.001
Provincial pooling → Level of medical institution visited	0.365	0.175	0.094	3.866	< 0.001
Level of medical institution visited → Health	0.071	0.060	0.046	1.539	0.124

To analyze the mechanism of health insurance provincial pooling on participants' health further, this study decomposed specific paths and presented the relevant results in Table 6. The results showed that the direct effect accounted for 87.9% of the total effect, while the indirect effect accounted for 12.1% of the total effect. The 95% confidence interval of the total effect, direct effect, and indirect effect did not contain 0, indicating significant mediating effects. Specifically, medical cost burden serves as a mediator in the relationship between provincial pooling and health.

**Table 6:** Results of decomposition of mediating effects of medical cost burden

Path effect	Impact effect	Standard error	t value	95%CI		P	Effect ratio
				Lower limit	Upper limit		
Total effect	1.073	0.102	10.491	0.8724	1.2746	0.000	100%
Direct effect	0.944	0.099	9.467	0.7484	1.1405	0.000	87.9%
Indirect effect	0.129	0.042	-	0.0519	0.2205	-	12.1%

### Heterogeneity analysis

To further validate the heterogeneity of effects resulting from the implementation of the provincial pooling policy for basic medical insurance, this paper examines the policy's impact on medical cost burden, medical service utilization, and health for participants. The analysis is structured across two dimensions: income and age. The sections below elaborate on the findings.

Firstly, the heterogeneous effects of different income levels are analyzed. Table 7 displays the effects of the provincial pooling policy for basic medical insurance on medical cost burden, medical service utilization, and health for high, middle, and low-income participants. The policy has a negative impact on the medical cost burden for participants at all income levels, with a significance level of 1%. However, the policy variable coefficient is the smallest for low-income participants (-0.2273), followed by middle-income (-0.2091) and high-income participants (-0.1125). This suggests that the provincial health insurance policy is more effective in reducing the medical cost burden for low-income participants. Regarding medical institution levels, the policy's impact varies among different income levels, but there is a significant positive effect only for low-income participants (*p* < 0.01). This indicates that the policy is more beneficial in increasing access to higher-level medical institutions for low-income individuals. In terms of health, the provincial integration policy has a positive impact on middle and high-income participants' health, passing the significance test at the 1% level. However, the policy variable coefficient is greater for high-income participants compared to middle-income participants (1.7984 > 1.1836). These results show that the provincial integration policy helps to enhance the health of middle and high-income participants, with greater benefits for those in the high-income bracket.

**Table 7:** Income heterogeneity in the implementation effect of provincial pooling of basic medical insurance

Variables	Medical cost burden	Level of medical institution visited	Health
Low income			
Provincial pooling	-0.2273^b^(0.0255)	4.0875 ^b^(0.7496)	2.2174(0.8852)
Control variables	Control	Control	Control
Constant	0.0098(0.2920)	29.7183(24.2536)	1.2936(18.9421)
Time fixed effect	Control	Control	Control
Provincial fixed effect	Control	Control	Control
Observation	3572	3572	3572
R^2^	0.8746	0.9271	0.5305
Middle Income			
Provincial pooling	-0.2091^b^(0.0510)	1.8633(0.4537)	1.1836^b^(0.6862)
Control variables	Control	Control	Control
Constant	0.1493(0.5881)	2.8601(4.8674)	2.6720^b^(7.8500)
Time fixed effect	Control	Control	Control
Provincial fixed effect	Control	Control	Control
Observation	713	713	713
R^2^	0.9195	0.9059	0.6563
High income			
Provincial pooling	-0.1121^b^(0.0346)	1.3325(0.6014)	1.7984^b^(1.3980)
Control variables	Control	Control	Control
Constant	1.2314^a^(0.7559)	-5.9874(6.7022)	1.6720(16.1406)
Time fixed effect	Control	Control	Control
Provincial fixed effect	Control	Control	Control
Observation	1399	1399	1399
R^2^	0.7944	0.9378	0.7875

Note: Standard errors are in parentheses; ^b^, ^a^ denote significant at the 1%, and 10% levels of significance

The second is the heterogeneity effect analysis of different age groups. Table 8 exhibits the effects of the provincial pooling policy for basic medical insurance on medical cost burden, medical service utilization, and health across high, middle, and low-age groups of insured individuals. The policy has a negative impact on the medical expense burden for all age groups, with significance at the 1% level. However, the smallest policy variable coefficient is observed for the high-age participants (-0.2710), demonstrating that the provincial pooling policy is more effective in reducing the medical expense burden for this age group.In terms of medical institution levels, the policy has a significant positive effect only for the elderly insured group (*P* < 0.01), indicating that the policy is more beneficial in upgrading the medical institution levels for elderly insured individuals.Regarding health, the policy has positive effects on middle and high-age participants' health, passing the significance test of 1%. These findings suggest that the provincial pooling policy fosters improved health in middle-aged and elderly participants.

**Table 8:** Age heterogeneity in the implementation effect of provincial pooling of basic medical insurance

Variables	Medical cost burden	Level of medical institution visited	Health
Lower age			
Provincial pooling	-0.1090^a^ (0.0008)	0.2299(0.2513)	1.9940 (0.3580)
Control variables	Control	Control	Control
Constant	0.1030 (0.0062)	1.3044 (1.5598)	2.217 (2.7610)
Time fixed effect	Control	Control	Control
Provincial fixed effect	Control	Control	Control
Observation	1427	1427	1427
R^2^	0.5327	0.7435	0.8848
Middle age			
Provincial pooling	-0.1190^a^ (0.0154)	0.1872 (0.1360)	1.9220^a^ (0.5310)
Control variables	Control	Control	Control
Constant	0.2380 (0.2980)	1.6940^a^ (1.5100)	1.004 (10.2300)
Time fixed effect	Control	Control	Control
Provincial fixed effect	Control	Control	Control
Observation	2383	2383	2383
R^2^	0.7922	0.6979	0.5238
Senior age			
Provincial pooling	-0.2710^a^ (0.0262)	1.9010^a^ (0.2432)	0.5900^a^ (0.5990)
Control variables	Control	Control	Control
Constant	0.0538 (0.2820)	0.0446 (2.4910)	3.6730 (6.4430)
Time fixed effect	Control	Control	Control
Provincial fixed effect	Control	Control	Control
Observation	1874	1874	1874
R^2^	0.9744	0.9734	0.5636

Note: Standard errors are in parentheses; ^a^ denote significant at the 1% levels of significance

### Further analysis

#### Differences in policy effects of different health insurance provincial pooling models

For a more comprehensive understanding, we first compare and analyze the impact of various pooling modes for medical insurance funds at the provincial level. Table 9 displays the findings. The unified income and expenditure mode and the risk adjustment fund mode both have negative impacts on insured individuals' medical cost burden (significant at the 1% level). However, the unified income and expenditure mode has a smaller impact coefficient compared to the risk adjustment fund mode (-0.2053 < -0.0775), suggesting that the two models have notably divergent effects on insured individuals' medical cost burden.

**Table 9:** Differences in policy effects of different health insurance provincial pooling models

Variables	Medical cost burden		Level of medical institution visited		Health	
Provincial-level unified revenue and expenditure model	-0.2053^c^ (0.0059)		1.8552^c^ (0.5190)		2.8406^c^ (0.1353)	
Provincial risk adjustment fund model		-0.0775^c^ (0.0055)		0.8878^c^ (0.1525)		0.6812^b^ (0.1290)
Age	0.0048^c^ (0.0014)	0.0014 (0.0013)	0.0123 (0.0306)	0.0051 (0.0282)	0.0793^b^ (0.0310)	0.0483 (0.0314)
Gender	-0.0041^c^ (0.0014)	-0.0032^b^ (0.0013)	-0.0537 (0.0402)	-0.0498 (0.0370)	-0.0332 (0.0318)	-0.0251 (0.0316)
Education	0.0006 (0.0007)	0.0010 (0.0007)	0.0493^c^ (0.0174)	0.0449^c^ (0.0161)	-0.0441^c^ (0.0166)	-0.0401^b^ (0.0165)
Marriage	0.0001 (0.0018)	0.0011 (0.0017)	-0.0131 (0.0508)	-0.0103 (0.0468)	-0.0039 (0.0409)	0.0051 (0.0406)
Income	0.0017^a^ (0.0009)	0.0018^b^ (0.0009)	-0.0093 (0.0229)	-0.0127 (0.0212)	-0.0223 (0.0206)	-0.0209 (0.0204)
Smoking Habit	0.0031^a^ (0.0016)	0.0026^a^ (0.0015)	0.0231 (0.0438)	0.0292 (0.0404)	-0.0133 (0.0362)	-0.0170 (0.0360)
Drinking habit	0.0025 (0.0016)	0.0028^a^ (0.0015)	-0.0124 (0.0390)	-0.0094 (0.0359)	0.0324 (0.0364)	0.0350 (0.0361)
Residence	0.0481^c^ (0.0011)	0.0540^c^ (0.0011)	-0.0541^c^ (0.0196)	-0.0303 (0.0185)	0.2549^c^ (0.0245)	0.3083^c^ (0.0263)
Economic level	-0.0247^b^ (0.0224)	-0.0185^c^ (0.0214)	0.0279 (0.3721)	0.0259 (0.3430)	-0.2725 (0.5131)	-0.2218 (0.5097)
Disease	0.0088^c^ (0.0021)	0.0059^c^ (0.0020)	0.0407 (0.0339)	0.0203 (0.0314)	-0.2590^c^ (0.0485)	-0.2340^c^ (0.0484)
Constant	0.3440 (0.2601)	0.2328 (0.2237)	0.7303 (3.8762)	0.7989 (3.6688)	4.5849 (5.8099)	6.6940 (5.8691)
Time fixed effect	Control	Control	Control	Control	Control	Control
Provincial fixed effect	Control	Control	Control	Control	Control	Control
Observation	5684	5684	5684	5684	5684	5684
R^2^	0.877	0.889	0.926	0.938	0.862	0.864

Note: Standard errors are in parentheses; ^c^, ^b^, ^a^ denote significant at the 1%, 5%, and 10% levels of significance

In terms of medical institution level, the two models have a positive impact on insured individuals' medical treatment quality. However, the impact coefficient for the unified income and expenditure mode is larger than for the risk adjustment fund mode (1.8552 > 0.8878), significant at the 1% level. This result indicates that the unified income and expenditure mode is more advantageous in providing insured individuals with access to superior medical institutions. In the health aspect, similar results emerge. Namely, the unified income and expenditure mode has a greater positive effect on health compared to the risk adjustment fund mode (2.8406 > 0.6812), with significance at a minimum level of 5%.

### Moral hazard in provincial pooling of basic medical insurance

In a mediating mechanism analysis, we find that provincial pooling of basic health insurance improves participants' access to medical institutions but does not enhance their health. This paper hypothesizes that this may be due to moral hazards, such as the excessive use of medical care by participants and induced demand by medical institutions.

To illustrate the existence of these moral hazards in provincial pooling, this paper further analyzes the impact of provincial pooling on participants' utilization of outpatient and inpatient services. Table 10 presents the results, showing that provincial pooling significantly and positively affects the utilization of outpatient and inpatient services. This indicates that the policy improves the use of medical services for participants. Simultaneously, the impact of provincial pooling on doctors requesting hospitalization is significantly positive, suggesting that medical institutions provide more inpatient services at higher pooling levels. Consequently, participants may be more inclined to use additional medical services, and medical institutions may be more interested in increasing their provisions.

**Table 10:** Moral hazard in the provincial pooling of basic medical insurance

Variables	Outpatient or not	Hospitalization or not	Does the doctor require hospitalization
Provincial pooling	0.9976^c^ (0.1602)	0.9984^c^ (0.0567)	0.9634^c^ (0.1363)
Age	0.0683^c^ (0.0141)	-0.0153 ^c^ (0.0054)	0.0050 (0.0115)
Gender	0.0116 (0.0159)	0.0028 (0.0059)	-0.0095 (0.0108)
Education	-0.0309 ^c^ (0.0097)	0.0109 ^c^ (0.0039)	-0.0066 (0.0058)
Marriage	-0.0368^a^ (0.0201)	0.0013 (0.0074)	-0.0138 (0.0141)
Income	-0.0010 (0.0104)	-0.0065 ^a^ (0.0038)	-0.0055 (0.0070)
Smoking Habit	-0.0220 (0.0173)	0.0032 (0.0064)	0.0204 ^a^ (0.0122)
Drinking habit	-0.0222 (0.0174)	0.0076 (0.0064)	0.0042 (0.0122)
Residence	-0.0386^b^ (0.0168)	0.0351^c^ (0.0105)	0.4119^c^ (0.0086)
Economic level	0.1735 (0.2932)	-0.0605 (0.1057)	0.4837^c^ (0.1797)
Disease	0.3103^c^ (0.0197)	0.0072 (0.0094)	-0.0870^c^ (0.0167)
Constant	0.8561 (3.4747)	0.5857 (1.2526)	5.3834^c^ (1.8814)
Time fixed effect	Control	Control	Control
Provincial fixed effect	Control	Control	Control
Observation	1070	989	1800
R^2^	0.866	0.981	0.856

Note: Standard errors are in parentheses; ^c^, ^b^, ^a^ denote significant at the 1%, 5%, and 10% levels of significance

## Discussion

### Provincial pooling of basic medical insurance has an impact on the health of participants

This paper aims to analyze the impact of the provincial pooling of basic health insurance on participants' health and its underlying mechanisms. The research findings reveal that provincial pooling improves participants' health, reduces their medical cost burden, and enhances the level of medical care they receive. These findings are consistent with existing research [17]. However, prior studies did not explore how provincial pooling affects health; this paper addresses this gap. Our mediating mechanism analysis indicates that provincial pooling enhances health by minimizing participants' medical cost burden but fails to improve health by increasing access to medical institutions.When the level of medical insurance pooling is raised, the unified medical insurance policy makes the treatment level more equitable between regions, and the increased protective treatment helps reduce the medical burden of participants. Reduced medical cost burdens imply lower future healthcare utilization expenses [18], which enhances participants' security expectations and alleviates worries about the financial burden caused by disease risk, ultimately leading to improved health [19]. Participants may also adjust their precautionary savings and consumption in other areas, resulting in enhanced health through a higher standard of living [20].

The observed lack of health improvement due to increased access to higher-ranking medical institutions may be attributable to moral hazards on both supply and demand sides. When basic health insurance is integrated at the provincial level, local health insurance agencies will have a greater incentive to loosen their supervision of the behavior of participants and medical institutions to obtain more provincial funds, as this will raise medical costs and expand health insurance fund expenditures. Under such circumstances, participants' medical referrals and reimbursement of medical expenses will not be strictly supervised, which will make participants more inclined to choose high-grade hospitals with better resources for treatment and thus overuse medical services. The decrease in the quality of supervision of medical institutions by health insurance agencies will make it difficult to discipline medical services, and the result will be an increase in induced demand without an increase in health standards.

Therefore, when promoting the upgrade of basic health insurance pooling levels, it is crucial not only to recognize the positive effects of provincial pooling on health but to address the moral hazard problems associated with diminished supervisory motivation from local health insurance institutions. In a provincial pooling framework, incentive and restraint mechanisms, as well as responsibility-sharing mechanisms between provincial and municipal levels, can be enhanced to strengthen local health insurance institutions' supervisory motivation. It is also essential to consolidate the hierarchical diagnosis and treatment system and improve primary medical institutions' service capacity and medical insurance reimbursement treatment. These measures will encourage participants to use medical resources rationally and curb excessive consumption of medical services.

### Heterogeneity in the impact of provincial pooling of basic health insurance on health

The findings in this paper suggest that there is heterogeneity in the effects of provincial pooling on health care cost burden, medical service utilization, and health. When analyzed at the income level, the provincial pooling of health insurance is more helpful in reducing the medical cost burden of low-income enrollees and improving the level of access to healthcare facilities, but more helpful in improving the health of middle and high-income earners.Income is an important factor influencing participants' medical behavior decisions and health, and low-income people are more likely to be unable to obtain necessary services on time due to financial burdens [21].

The increase in the level of health insurance benefits after provincial integration can effectively improve the sensitivity of low-income people to health care costs, which in turn has a more significant impact on their cost burden reduction and health service utilization. At the same time, the analysis results suggest that the protection scope for low-income groups should be further improved in provincial pooling. This is because provincial health insurance pooling aims to expand the risk-sharing scope and optimally use health insurance funds to protect vulnerable groups. However, the fact that the policy has no significant impact on low-income participants' health indicates the need to continually minimize their medical cost burden and enhance their disease risk resilience throughout the pooling level upgrade.

Analyzed at the age level, the provincial pooling of health insurance is more helpful in reducing the burden of medical costs and improving the health level of middle and older age participants, as well as in improving the level of access to healthcare facilities for participants in the older age groups. This may be because middle- and old-aged enrollees are in the middle and late stages of their life course, which is the period when previous latent health problems explode [22], and they are also more vulnerable to disease shocks and thus face higher health risks. In contrast, the provincial pooling of health insurance reduces the burden of health care costs for middle- and high-age participants by increasing the level of coverage, which in turn has a greater impact on their health care utilization and health.

### Different health insurance provincial pooling models can have a differential impact on health

A comparison of the effects of the unified revenue and expenditure model and the risk adjustment fund model reveals that the unified revenue and expenditure model is more effective in reducing the insured's medical cost burden and has a more positive impact on both medical institutions and health. This difference may be attributable to variations in fundraising and resource allocation scales under different provincial pooling modes.Under the unified revenue and expenditure model, local medical insurance institutions transfer the entire sum of medical insurance funds to the provincial level, where provincial medical insurance institutions manage unified allocation. In contrast, under the risk adjustment fund model, local governments transfer only a specific percentage of fund income to the provincial level, while the remaining funds are allocated by local governments. This demonstrates that the unified income and expenditure mode can harness larger-scale fundraising and facilitate inter-regional fund mutual aid, better utilizing medical insurance funds' "law of large numbers."

Additionally, the impact of different pooling modes on the medical cost burden shows that the unified income and expenditure mode has a more substantial negative effect on the medical cost burden related to economic factors. Currently, most provinces that have implemented the unified revenue and expenditure mode of basic medical insurance belong to regions with high economic development levels, such as Shanghai and Beijing. In contrast, regions with relatively lower economic development levels, like Hainan and Ningxia, implement the provincial risk adjustment fund model. Regions with better economic development tend to have more substantial financing and treatment levels in basic medical insurance compared to those with lower economic development levels, thus providing better protection for the insured.

Considering the advantages of the unified revenue and expenditure provincial pooling model, some studies suggest that the provincial risk adjustment fund model is a transitional stage in promoting medical insurance pooling level improvements, while the unified revenue and expenditure model represents the long-term goal [23]. Depending on their medical insurance development and economic development levels, different regions can choose suitable provincial pooling modes. If the differences between provincial regions are minimal, the unified income and expenditure model is appropriate. If significant differences exist, the risk adjustment fund can be adopted in the early stages of pooling. As regional disparities gradually reduce, transitioning to the unified income and expenditure model can help lessen resistance to provincial pooling policy implementation and steadfastly achieve the goal of enhancing medical insurance pooling levels.

### Robustness test

The findings in this paper indicate that the provincial pooling policy for basic medical insurance impacts participants' medical cost burden, medical service utilization, and health. Nonetheless, a series of robustness tests are needed to eliminate confounding factors. In this paper, we analyze various aspects such as the parallel trend test, PSM-DID model, placebo test, and sample tailing treatment to ensure the estimation results' robustness. All the above robustness analyses indicate that the results of this paper are stable, and the specific robustness test results are shown in Additional file 1.

## Conclusion

In summary, this paper concludes that provincial pooling of basic medical insurance can reduce participants' medical cost burden, increase the level of medical institutions, and improve health. Provincial pooling positively impacts participants' health primarily by reducing medical cost burdens. However, due to the moral hazard for both medical institutions and participants, provincial pooling fails to enhance health by increasing medical institution levels. Therefore, the targeted prevention of moral risks of excessive medical care for participants and induced demand by medical institutions, as well as the improvement of incentive and restraint mechanisms between provincial and municipal medical insurance institutions, are the essential focal points in refining the provincial pooling policy for basic medical insurance.

### Limitations

Due to data limitations, it was challenging to obtain quantitative indicators directly reflecting the supervisory motivation of local health insurance agencies in obtaining more provincial integration funds after provincial pooling.To address the moral hazard of participants and medical institutions in provincial pooling, this paper used relevant health service utilization indicators for preliminary verification.However, future research could benefit from incorporating new effective variables to provide a more comprehensive explanation of self-interest behavior among different subjects in provincial health insurance pooling.

Moreover, this study used self-assessed health indicators to reflect individuals' health status, but these measures are subjective. Therefore, future research should focus on enriching and improving measurement indicators. Additionally, the proxy variable used to represent the type of illness among participants was limited to the question "Have you had any illness or injury in the past two weeks?" While it provides some insight into participants' illnesses, it does not capture the specific types of illnesses.

Furthermore, the classification of cities where participants reside aimed to reflect differences between locations to some extent. However, it has limitations as it does not capture more detailed variations between different places of residence. In future studies, considering a more detailed division into urban and rural areas could provide insights into the possible effects of participants' residential differences.

Overall, the study acknowledges its limitations and highlights potential avenues for future research to overcome these constraints and improve the understanding of provincial health insurance pooling.

## Supplementary Information

**Additional file 1.** Robustness analysis.

## Data Availability

Please contact author for data requests.
