# Supplementary material for: The promotion of pooling level of basic medical insurance and participants’ health: impact effects and mediating mechanisms
Source: Int J Equity Health. 2023 Jun 7;22:113. doi: 10.1186/s12939-023-01927-1 (PMC10246363; doi:10.1186/s12939-023-01927-1)
Supplement: Supplementary file 1 — Additional file 1. Robustness analysis. [file 12939_2023_1927_MOESM1_ESM.doc]

**Robustness analysis**

The regression results suggest the provincial coordination policy of basic health insurance impacts participants' healthcare utilization and health. Nonetheless, a series of robustness tests are required to isolate confounding factors from the study findings. In this paper, we examine several dimensions such as parallel trend tests, PSM-DID models, placebo tests, and sample tailing treatments to ensure the estimation results' robustness, with specific robustness test results analyzed as follows.

**Parallel trend test**

As the double difference model necessitates satisfying the "parallel trend assumption," we use dynamic effects analysis based on the event analysis method for robustness testing to assess the empirical results' sturdiness. The results are depicted in Figure 1. Period 0 on the horizontal axis signifies the current period of the basic medical insurance policy's provincial-level implementation, while periods -1 and 1 denote the policy implementation's preceding and subsequent phases, respectively. The vertical axis represents the average treatment effect produced by the provincial coordination policy's enforcement. It is evident that there is no significant distinction in participants' health levels between the provincial coordination group and the non-uniformed group before implementing the provincial coordination policy, indicating that the parallel trend assumption is met.


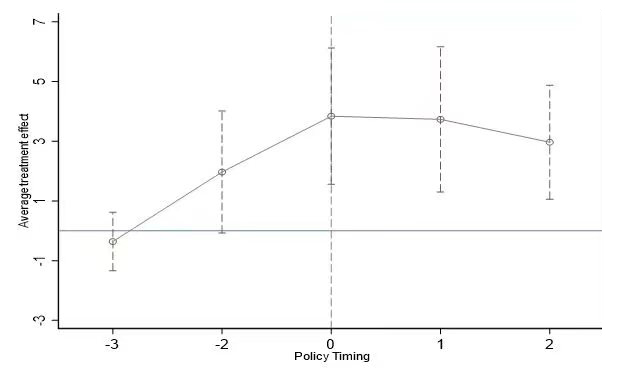


**Figure 1** Parallel trend test

**Psm-did**

Considering the systematic errors introduced by unobservable variables, we further employ the PSM-DID model to re-estimate the provincial coordination of basic health insurance's impact on healthcare utilization and health. PSM is instrumental in reducing the correlation between treatment and observable variables, mitigating selectivity bias by controlling for observable variables correlated with the explanatory and treatment variables. Table 1 displays the regression results obtained using the PSM-DID model. After accounting for relevant variables, the regression coefficients of provincial coordination variables are largely consistent with the earlier regression results, indicating the results' robustness.

**Table 1** PSM-DID estimation results

| Variables | Medical Cost Burden | Level of medical institution visited | Health |
| --- | --- | --- | --- |
| Provincial pooling | -0.1383*  (0.0824) | 2.7224***  (0.6095) | 3.6839**  (1.5842) |
| Age | 0.0107  (0.0290) | 0.1289  (0.2148) | -0.0757  (0.5583) |
| Gender | -0.0080  (0.0060) | -0.0555  (0.0446) | -0.0201  (0.1158) |
| Education | 0.0071**  (0.0028) | 0.0452**  (0.0210) | -0.0092  (0.0547) |
| Marriage | 0.0091  (0.0094) | -0.0580  (0.0695) | 0.2507  (0.1807) |
| Income | 0.0086**  (0.0036) | -0.0305  (0.0268) | -0.1282*  (0.0696) |
| Smoking Habit | 0.0027  (0.0065) | -0.0287  (0.0483) | -0.1989  (0.1255) |
| Drinking habit | 0.0041  (0.0060) | 0.0085  (0.0445) | 0.0749  (0.1157) |
| Residence | 0.0014  (0.0146) | 0.2183  (0.2803) | 0.2183  (0.2803) |
| Economic level | 0.0259  (0.0512) | -0.3532  (0.9852) | -0.3532  (0.9852) |
| Disease | 0.0066  (0.0054) | 0.1682  (0.1031) | 0.1682  (0.1031) |
| Constant | 0.2069  (0.5359) | 2.0450  (3.9662) | 5.0774  (10.3082) |
| Time fixed effect | Control | Control | Control |
| Provincial fixed effect | Control | Control | Control |
| Observation | 5128 | 5128 | 5128 |
| R^2^ | 0.884 | 0.811 | 0.639 |

Note: Standard errors are in parentheses; ***, **, * denote significant at the 1%, 5%, and 10% levels

of significance

**Placebo test**

Although the double difference method can minimize the factors requiring control in the model through the inclusion of control group controls, and we incorporated province and time-fixed effects in the basic test to lessen the omitted variable problem's interference on the results, controlling for certain factors co-varying with location and time may still be challenging. Based on this, we perform a robustness analysis using a placebo test, which involves random selection of the "provincial coordination policy" pilot provinces, random generation of reform years, construction of randomized experiments at the reform year and province levels, and subsequent regression based on the aforementioned econometric model. We evaluate the findings' reliability based on the probability of obtaining the baseline regressions' estimated coefficients from the spurious experiments. To further bolster the placebo test's validity, we repeated the aforementioned process 500 times and ultimately plotted the distribution of estimated coefficients. If the distribution of estimated coefficients under the randomized treatment is around 0, it suggests that no significantly important influences were omitted from the model set. As observed from the distribution of estimated coefficients presented in Figure 2, the spurious double difference term's estimated coefficients are centered around 0, indicating the model setting has no severe problem with omitted variables and the core findings remain robust.


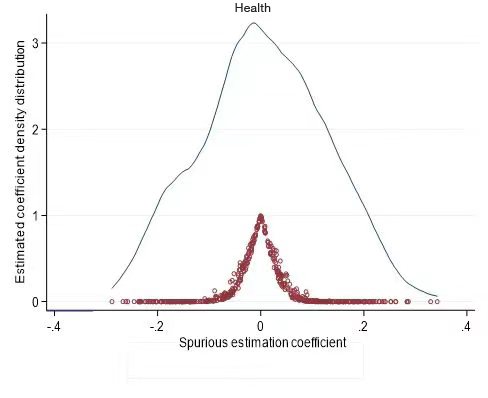


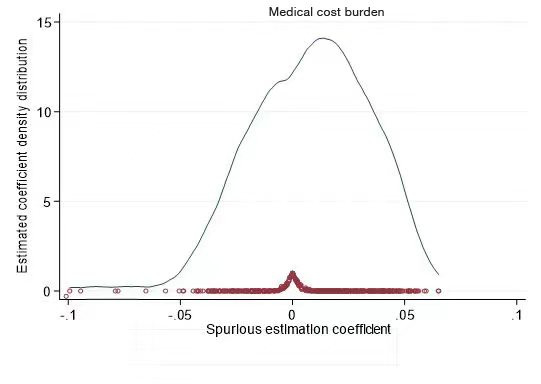


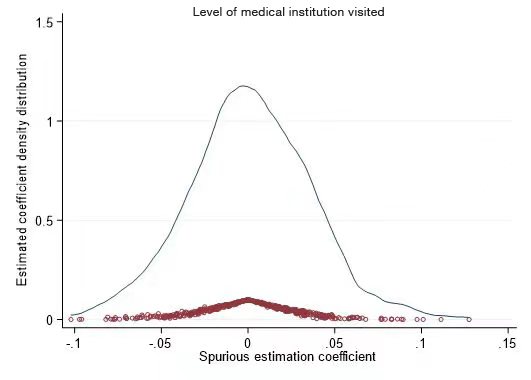


**Figure 2** Placebo test

**Sample tailing process**

To avoid the influence of extreme values on the analysis results, this paper further adopts the sample tailing process for robustness testing, i.e., replacing each variable less than or greater than 1% and 99% with the quantile corresponding to 1% and 99%, and then running a new regression, and the results are shown in Table 2. It can be seen that the coefficients of the effect of provincial coordination policy on health care utilization and health all pass the significance test at the 1% level, indicating that the estimation results are robust.

**Table 2** Results of sample shrinkage tail robustness estimation

| Variables | Medical Cost Burden | Level of medical institution visited | Health |
| --- | --- | --- | --- |
| Provincial pooling | -0.1210***  (0.0181) | 1.7221***  (0.4441) | 2.8989***  (0.4140) |
| Age | 0.0046***  (0.0013) | 0.0113  (0.0307) | 0.0820***  (0.0306) |
| Gender | -0.0040***  (0.0014) | -0.0509  (0.0400) | -0.0353  (0.0314) |
| Education | 0.0004  (0.0007) | 0.0476***  (0.0177) | -0.0450***  (0.0164) |
| Marriage | 0.0003  (0.0018) | -0.0135  (0.0508) | -0.0071  (0.0404) |
| Income | 0.0011  (0.0008) | -0.0021  (0.0234) | 0.0033  (0.0189) |
| Smoking Habit | 0.0029*  (0.0016) | 0.0204  (0.0438) | -0.0088  (0.0358) |
| Drinking habit | 0.0022  (0.0016) | -0.0130  (0.0390) | 0.0377  (0.0360) |
| Residence | 0.0480***  (0.0011) | -0.0537***  (0.0195) | 0.2574***  (0.0242) |
| Economic level | -0.0307  (0.0221) | 0.0687  (0.3620) | -0.1601  (0.5044) |
| Disease | 0.0087***  (0.0021) | 0.0416  (0.0339) | -0.2598***  (0.0479) |
| Constant | 0.3280  (0.2616) | 0.3939  (3.8740) | 3.1551  (5.9594) |
| Time fixed effect | Control | Control | Control |
| Provincial fixed effect | Control | Control | Control |
| Observation | 5684 | 5684 | 5684 |
| R^2^ | 0.880 | 0.865 | 0.926 |

Note: Standard errors are in parentheses; ***, **, * denote significant at the 1%, 5%, and 10%

levels of significance
